# HMGB1 Modulates High Glucose-Induced Erroneous Differentiation of Tendon Stem/Progenitor Cells through RAGE/*β*-Catenin Pathway

**DOI:** 10.1155/2024/2335270

**Published:** 2024-04-09

**Authors:** Panpan Lu, Guangchun Dai, Liu Shi, Yingjuan Li, Ming Zhang, Hao Wang, Yunfeng Rui

**Affiliations:** ^1^Department of Orthopaedics, Zhongda Hospital, Southeast University, No 87 Ding Jia Qiao, Nanjing 210009, Jiangsu, China; ^2^School of Medicine, Southeast University, No 87 Ding Jia Qiao, Nanjing 210009, Jiangsu, China; ^3^Orthopaedic Trauma Institute (OTI), Southeast University, No 87 Ding Jia Qiao, Nanjing 210009, Jiangsu, China; ^4^Trauma Center, Zhongda Hospital, Southeast University, No 87 Ding Jia Qiao, Nanjing 210009, Jiangsu, China; ^5^Department of Geriatrics, Zhongda Hospital, Southeast University, No 87 Ding Jia Qiao, Nanjing 210009, Jiangsu, China

## Abstract

The association of tendinopathy with diabetes has been well recognized. Tendon stem/progenitor cells (TSPCs) play critical roles in tendon repair, regeneration, and homeostasis maintenance. Diabetic TSPCs exhibit enhanced erroneous differentiation and are involved in the pathogenesis of diabetic tendinopathy, whereas the underlying mechanism of the erroneous differentiation of TSPCs remains unclear. Here, we showed that high glucose treatment promoted the erroneous differentiation of TSPCs with increased osteogenic differentiation capacity and decreased tenogenic differentiation ability, and stimulated the expression and further secretion of HMGB1 in TSPCs and. Functionally, exogenous HMGB1 significantly enhanced the erroneous differentiation of TSPCs, while HMGB1 knockdown mitigated high glucose-promoted erroneous differentiation of TSPCs. Mechanistically, the RAGE/*β*-catenin signaling was activated in TSPCs under high glucose, and HMGB1 knockdown inhibited the activity of RAGE/*β*-catenin signaling. Inhibition of RAGE/*β*-catenin signaling could ameliorate high glucose-induced erroneous differentiation of TSPCs. These results indicated that HMGB1 regulated high glucose-induced erroneous differentiation of TSPCs through the RAGE/*β*-catenin signaling pathway. Collectively, our findings suggest a novel essential mechanism of the erroneous differentiation of TSPCs, which might contribute to the pathogenesis of diabetic tendinopathy and provide a promising therapeutic target and approach for diabetic tendinopathy.

## 1. Introduction

Tendinopathy, characterized by chronic pain, tendon rupture, declined exercise tolerance, and reduced mobility, describes a complex and multifactorial pathology [1]. In addition to overuse or excess load, there is strong evidence that diabetes mellitus is significantly associated with higher risk of tendinopathy [2]. In diabetic tendinopathy, the structural and functional properties of the tendon are compromised, and the tendon becomes more susceptible to degeneration and subsequent injury or rupture [3, 4]. In addition, the level of tenogenic differentiation genes is decreased in diabetic tendons, whereas the expression of osteo-chondrogenic differentiation genes is significantly increased [5, 6]. Recently, hyperglycemia has been shown to contribute to the development of diabetic tendinopathy by leading to cellular damage, inducing oxidative stress, altering extracellular matrix, and impairing tendon homeostasis [7, 8]. However, the pathogenesis of diabetic tendinopathy has not been fully elucidated [3, 9]. Due to the limited capacity for endogenous regeneration and repair, it is difficult to restore the original properties of the injured tendons with existing medical treatment strategies. Tendon stem/progenitor cells (TSPCs) have been isolated and identified from various species [10, 11]. Due to the self-renewal and multilineage differentiation capacity, TSPCs have been demonstrated to play vital roles in tendon repair, regeneration, and homeostasis maintenance [12, 13]. However, our previous studies have shown that TSPCs isolated from diabetic rat tendons exhibit reduced self-renewal ability and erroneous differentiation with enhanced osteo-chondrogenic differentiation capacity and decreased tenogenic differentiation ability, demonstrating that TSPCs are involved in the pathogenesis of diabetic tendinopathy [5]. These changes in TSPCs under diabetic conditions may impair tendon healing and regeneration ability. Studies reported that high glucose treatment could inhibit the cell proliferation, reduce the expression of tenogenic differentiation markers, and increase the expression of osteo-chondrogenic differentiation markers in TSPCs [14], implying that the hyperglycemic microenvironment may play a vital role in the erroneous differentiation of TSPCs and diabetic tendinopathy. Nevertheless, the underlying mechanism of the erroneous differentiation of TSPCs in diabetic tendinopathy remains unclear.

High mobility group box 1 (HMGB1), a member of the high mobility group protein family and a widely expressed nuclear protein, has been reported to play vital roles in various biological and pathological processes, such as inflammation [15], differentiation, and migration [16]. However, HMGB1 can be secreted into the extracellular milieu in response to tissue damage or cellular stress, acts as a multifunctional cytokine to engage in tissue injury and regeneration [17, 18], and regulates several cellular processes, such as cell migration, differentiation, and proliferation [19]. Previous studies have indicated that HMGB1 is upregulated in diabetic patients and animal models and is involved in the pathogenesis of many diabetes-related diseases [19, 20]. Intriguingly, recent studies have also shown that HMGB1, as a damage-associated molecular pattern (DAMP) or an alarmin, is increased and plays significant roles in human [18, 21], mouse [22, 23], and rat [24, 25] tendinopathy, such as proinflammatory response and matrix regulation, suggesting that HMGB1 may be a potential new target for tendinopathy treatment [26]. In addition, previous studies have also indicated that HMGB1 is involved in the regulation of stem cells, promoting the proliferation, migration, and osteogenic differentiation of mesenchymal stem cells (MSCs) [27, 28]. The receptor for advanced glycation end-product (RAGE) is a functional receptor for HMGB1, to which HMGB1 can bind to regulate the proliferation, migration, and differentiation of stem cells [16]. Wang et al. [29] demonstrated that HMGB1 was functionally involved in the osteoblastic differentiation and calcification of human aortic valve interstitial cells through RAGE activation. In addition, intracellular *β*-catenin signaling has emerged as a critical regulator of the osteogenic differentiation of stem cells [30, 31]. In the nucleus, *β*-catenin is known to interact with transcription factor-4 (TCF-4) and activates the transcription of osteogenic genes [32]. Wang et al. [33] showed that HMGB1 exerted a pro-osteogenic effect on human bone marrow-derived MSCs (BMSCs) via activating the *β*-catenin signaling. Jin et al. [34] reported that HMGB1 could promote vascular calcification in chronic kidney disease through the *β*-catenin signaling. Although previous studies have indicated the vital role of HMGB1 in tendinopathy and in the regulation of stem cells, no studies have focused on the role of HMGB1 in TSPCs during diabetic tendinopathy.

In the present study, we investigated the potential role and mechanism of high glucose on the differentiation of TSPCs. We demonstrated that high glucose promoted the erroneous differentiation of TSPCs, and that the expression and secretion of HMGB1 were increased in TSPCs under high glucose treatment. Moreover, increased HMGB1 was associated with the activation of the RAGE/*β*-catenin signaling pathway, which was involved in high glucose-induced erroneous differentiation of TSPCs. Furthermore, both HMGB1 knockdown and inhibition of RAGE/*β*-catenin signaling ameliorated the erroneous differentiation of TSPCs. Our results collectively indicated a novel mechanism of the erroneous differentiation of TSPCs and provided a promising therapeutic target and approach for diabetic tendinopathy.

## 2. Materials and Methods

### 2.1. TSPCs Isolation and Culture

All the experiments were approved by the Animal Research Ethics Committee of Southeast University. Eight-week-old male Sprague–Dawley rats were used for TSPCs isolation and culture. The procedures used for TSPCs isolation and culture have been well-established in our previous studies [5, 10]. Briefly, the patellar tendons were minced and digested with type I collagenase (3 mg/ml, Sigma–Aldrich). The mixture was then passed through a 70 *μ*m cell strainer (Becton Dickinson) to yield a single-cell suspension. The cells were cultured in a complete medium containing low-glucose Dulbecco's modified Eagle's medium (DMEM), 10% fetal bovine serum, 100 U/ml penicillin, and 100 mg/ml streptomycin (all from Gibco). The cells were cultured at an optimal low density (50 nucleated cells/cm^2^) to isolate TSPCs, trypsinized, and mixed together as passage 0 (P0) [10]. TSPCs at P3–P5 were used for all the experiments. The clonogenicity and multilineage differentiation potential of TSPCs were confirmed by standard assays as described previously [10]. Additional D-glucose (Sigma–Aldrich) was added to the complete medium to prepare high glucose (15 and 25 mM) medium.

### 2.2. Osteogenic Differentiation Assay

The induction of osteogenic differentiation in TSPCs has been well-described in our previous studies [5, 10]. TSPCs were seeded in six-well plates at 4 × 10^3^ cells/cm^2^, cultured in complete medium until confluence, and subsequently cultured in complete basal medium (BM) or osteogenic induction medium (OIM), which was complete BM supplemented with 20 mM *β*-glycerolphosphate, 1 nM dexamethasone, and 50 mM ascorbic acid (all from Sigma–Aldrich) for 14 days. Quantitative real-time reverse transcription polymerase chain reaction (qRT-PCR) was used to detect the mRNA expression of the osteogenic genes runt-related transcription factor 2 (Runx2), osteopontin (OPN), and osteocalcin (OCN), and Alizarin red S (ARS) staining was performed to evaluate calcium nodule formation.

### 2.3. mRNA Expression of Tenogenic Genes

TSPCs were seeded in six-well plates at 4 × 10^3^ cells/cm^2^ and cultured in a complete medium. On day 7, the TSPCs were harvested, and the mRNA expression of the tenogenic genes collagen type I A 1 chain (Col 1A1), scleraxis (Scx), and tenomodulin (Tnmd) were measured by qRT-PCR.

### 2.4. qRT-PCR Assay

TSPCs were harvested and homogenized for total RNA extraction with TRIzol reagent (Invitrogen). The mRNA was reversely transcribed to cDNA using HiScript III 1st Strand cDNA Synthesis Kit (Vazyme) according to the manufacturer's instructions. One microliter of total cDNA from each sample was amplified in a 20 *μ*l reaction mixture containing ChamQ SYBR qPCR Master Mix (Vazyme) and specific primers for target genes using the ABI Step One Plus system (Applied Biosystems). The relative expression of each target gene was normalized to that of the *β*-actin gene and calculated as the fold change using the 2^−*ΔΔ*CT^ formula. The sequences of primers used in this study are listed in *Supplementary 1*.

### 2.5. Cell Transfection

The procedures for cell transfection were well-established in our previous study [35]. Lentiviruses targeting HMGB1 and negative control vectors were purchased from GeneChem Corporation (Shanghai, China). Briefly, 8 × 10^4^ cells were seeded in six-well plates. Cells at 20%–30% confluence were transfected with lentivirus using HitransG Transfection Reagent P (GeneChem) according to the manufacturer's instructions. The transfection efficiency was verified by qRT-PCR and western blot assays.

### 2.6. Western Blot Analysis

Total protein was extracted from the cells using Total Protein Extraction Kit or Nuclear and Cytoplasmic Protein Extraction Kit (Keygen Biotech) according to the manufacturer's protocols. The protein concentrations of the samples were detected by a BCA protein assay kit (Keygen Biotech). The protein was separated via SDS-PAGE and electro-transferred to PVDF membranes (Millipore). The membranes were blocked with 5% fat-free milk in TBST solution and subsequently incubated with primary antibodies at 4°C overnight. The primary antibodies are shown in *Supplementary 2*. After incubation with secondary antibodies (Proteintech) for 2 hr at room temperature, the blots were detected by enhanced chemiluminescence reagents (Keygen Biotech). The gray value of each band was examined, and the data were normalized to *β*-actin or Lamin B1.

### 2.7. Enzyme-Linked Immunosorbent Assay (ELISA)

The supernatant of each cell sample was collected and centrifuged at 1,000 *g* for 5 min to remove cell debris. An ELISA kit (Solarbio) was used to detect the levels of HMGB1 in each group according to the manufacturer's protocols.

### 2.8. Immunofluorescence Staining

TSPCs were fixed in 4% paraformaldehyde for 15 min, permeabilized with 0.2% Triton X-100 for 20 min, and then blocked with 3% bovine serum albumin for 1 hr at room temperature. The cells were washed and then incubated with primary antibodies at 4°C overnight, followed by incubation with a mixture of Alexa Fluor 594-conjugated goat anti-rabbit IgG (Cell Signaling Technology). The nuclei were counterstained with DAPI. Immunofluorescence was visualized under a Nikon fluorescence microscope.

### 2.9. Statistical Analysis

The statistical analysis was performed using SPSS version 16.0. All the data were presented as the mean ± standard deviation. Differences in mean values between groups were analyzed using one-way analysis of variance (ANOVA) followed by Tukey's post hoc multiple comparison tests. *P*  < 0.05 was considered statistically significant.

## 3. Results

### 3.1. High Glucose Treatment Promotes Erroneous Differentiation of TSPCs

**Figure 1:**
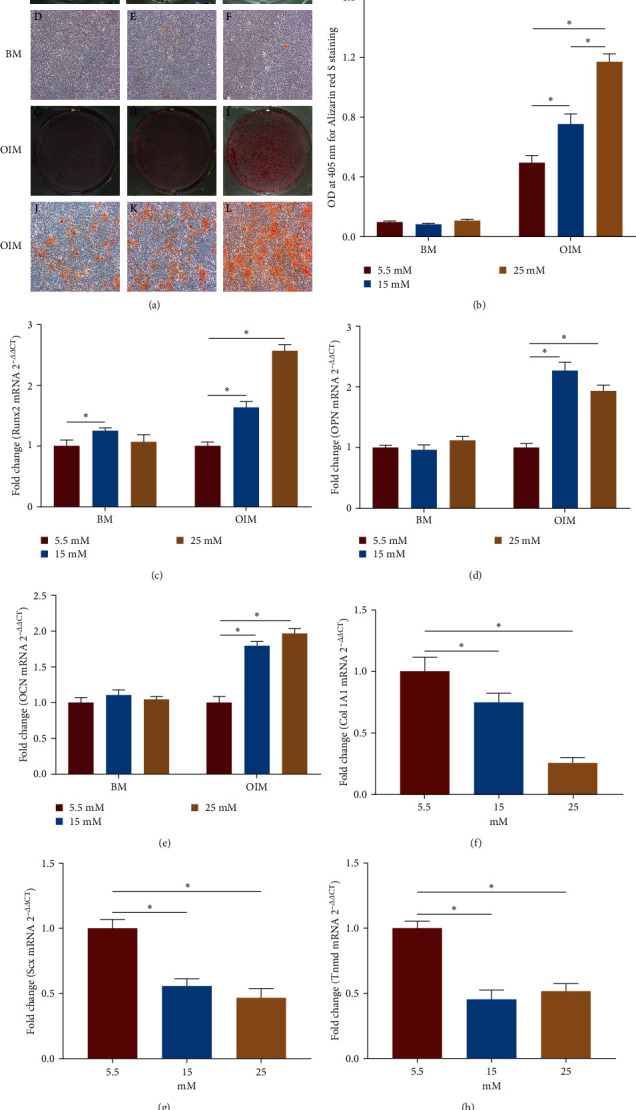
The osteogenic and tenogenic differentiation capacity of TSPCs under high glucose treatment. (a) Representative images of ARS staining of TSPCs in BM (A–F) and OIM (G–L) under normal glucose (5.5 mM) and high glucose (15 and 25 mM) on day 14. (b) Quantification of ARS bound to the calcium nodules in TSPCs. (c–e) Relative mRNA levels of osteogenic genes in TSPCs cultured in BM or OIM under normal glucose or high glucose for 14 days by qRT-PCR. (f–h) Relative mRNA levels of tendon-related genes in TSPCs by qRT-PCR. A–C, G–I × 1; D–F, J–L × 40.  ^*∗*^*P*  < 0.05.

First, we analyzed the effect of high glucose on the differentiation ability of TSPCs. As shown by ARS staining, distinct calcium nodules were observed in TSPCs cultured in OIM but not in BM (Figure 1(a)). Furthermore, more ARS-positive calcium nodules were observed in OIM under high glucose (15 and 25 mM) than under normal glucose (5.5 mM) (Figures 1(a), (G–L)). Quantification analysis showed that the intensity of calcium-bound ARS was greater in OIM under high glucose than under normal glucose (Figure 1(b)). Moreover, the mRNA levels of osteogenic genes, including Runx2, OPN, and OCN, were remarkably upregulated in TSPCs after osteogenic induction under high glucose compared with those under normal glucose, whereas there was no significant change in the mRNA expression of these genes in TSPCs in BM under high glucose but a slight increase in the expression of Runx2 (Figure 1(c)–1(e)). To investigate the effect of high glucose on the tenogenic differentiation ability of TSPCs, we further analyzed the expression of critical tendon-related genes, including Col 1A1, Scx, and Tnmd, in TSPCs. qRT-PCR showed that the mRNA levels of these markers were significantly downregulated in TSPCs treated with high glucose (Figure 1(f)–1(h)). These results indicated that high glucose treatment enhanced the osteogenic differentiation potential and reduced the tenogenic differentiation ability of TSPCs; that is, it promoted the erroneous differentiation of TSPCs.

### 3.2. HMGB1 Expression and Secretion Are Increased in TSPCs under High Glucose

**Figure 2:**
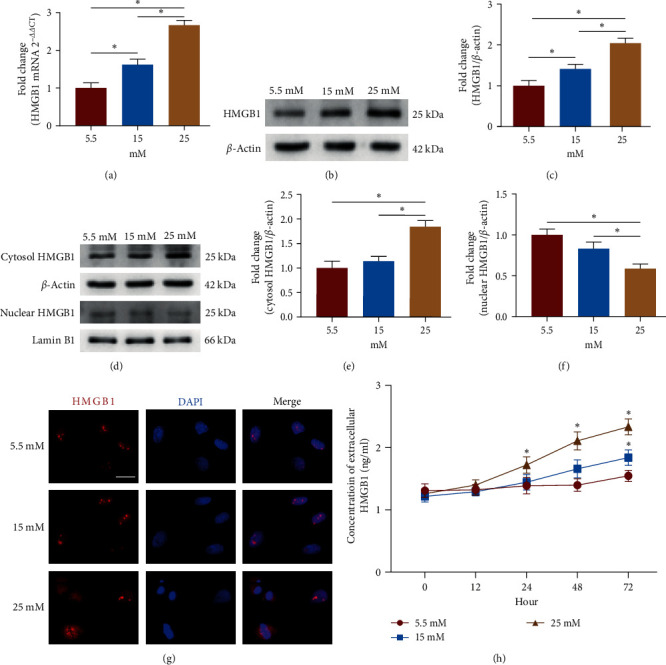
Increased expression and secretion of HMGB1 in TSPCs under high glucose. (a) Relative mRNA levels of HMGB1 in TSPCs by qRT-PCR. (b, c) Western blot analysis of HMGB1 protein expression in TSPCs. (d–f) Western blot analysis of HMGB1 protein levels in the nucleus and cytosol of TSPCs. (g) Immunofluorescence staining for HMGB1 in TSPCs. Scale bar: 50 *μ*m. (h) ELISA analysis of HMGB1 levels in the medium supernatant of TSPCs under normal glucose or high glucose.  ^*∗*^*P*  < 0.05.

Next, we examined the expression of HMGB1 in TSPCs. The mRNA and protein expression of HMGB1 was significantly increased in TSPCs under high glucose compared to normal glucose (Figure 2(a)–2(c)). Given the functional variability of HMGB1 at different locations [26], we then examined the subcellular distribution and levels of HMGB1 in TSPCs. The results showed that the level of nuclear HMGB1 was significantly decreased in TSPCs under high glucose treatment, while the level of cytoplasmic HMGB1 was notably elevated simultaneously (Figure 2(d)–2(f)). Immunofluorescence staining further revealed that HMGB1 was predominantly located in the nucleus of TSPCs cultured with normal glucose, whereas the level of HMGB1 distributed within the cytoplasm was significantly increased in TSPCs treated with high glucose (Figure 2(g)), indicating the promoted translocation of HMGB1 from the nucleus to the cytosol. Furthermore, we measured the extracellular HMGB1 in the culture supernatant by ELISA. The results showed that the concentration of HMGB1 in the medium supernatant under 25 mM of high glucose was significantly higher after 24 hr than under normal glucose, and the concentration of HMGB1 in the medium supernatant under 15 mM of high glucose was significantly higher after 72 hr than under normal glucose (Figure 2(h)), suggesting increased HMGB1 secretion under high glucose. These results demonstrated the increased expression and secretion of HMGB1 in TSPCs under high glucose.

### 3.3. Exogenous HMGB1 Enhances the Erroneous Differentiation of TSPCs

**Figure 3:**
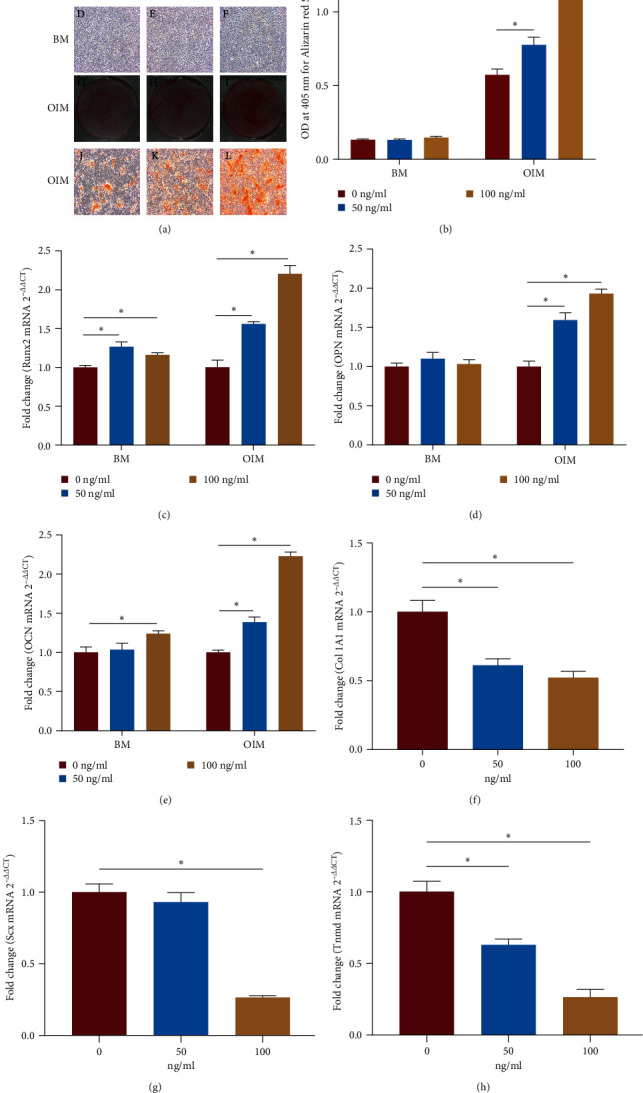
Exogenous HMGB1 promotes the erroneous differentiation of TSPCs. (a) Representative images of ARS staining of TSPCs in BM (A–F) and OIM (G–L) with different concentrations of recombinant HMGB1 (0, 50, and 100 ng/ml) on day 14. (b) Quantification of ARS bound to the calcium nodules in TSPCs. (c–e) Relative mRNA levels of osteogenic genes in TSPCs cultured in BM or OIM with different concentrations of recombinant HMGB1 on day 14 by qRT-PCR. (f–h) Relative mRNA levels of tendon-related genes in TSPCs treated with different concentrations of recombinant HMGB1 by qRT-PCR. A–C, G–I × 1; D–F, J–L × 40.  ^*∗*^*P*  < 0.05.

To explore the effect of HMGB1 on the differentiation of TSPCs, TSPCs were cultured in basal or osteogenic medium with different concentrations of recombinant HMGB1. As shown by ARS staining, significant calcium nodules were observed in TSPCs cultured in OIM with or without recombinant HMGB1 but not in the BM (Figure 3(a)). Moreover, the ARS-positive calcium nodules were significantly increased in OIM with recombinant HMGB1 (Figure 3(a), (G–L)). Quantification analysis showed that the intensity of calcium-bound ARS was higher in OIM containing recombinant HMGB1 than in OIM without recombinant HMGB1 (Figure 3(b)). Additionally, after recombinant HMGB1 treatment, the mRNA expression of the osteogenic genes Runx2, OPN, and OCN was significantly increased in TSPCs cultured in OIM, and the mRNA expression of Runx2 and OCN was increased in TSPCs cultured in BM (Figure 3(c)–3(e)). Nevertheless, the mRNA levels of the tenogenic genes Col 1A1, Scx, and Tnmd were significantly downregulated in TSPCs treated with recombinant HMGB1 (Figure 3(f)–3(h)), suggesting decreased tenogenic differentiation ability of TSPCs. These results indicated that exogenous HMGB1 promoted the erroneous differentiation of TSPCs, suggesting that HMGB1 might play a critical role in regulating the differentiation of TSPCs.

### 3.4. HMGB1 Knockdown Mitigates High Glucose-Induced Erroneous Differentiation of TSPCs

**Figure 4:**
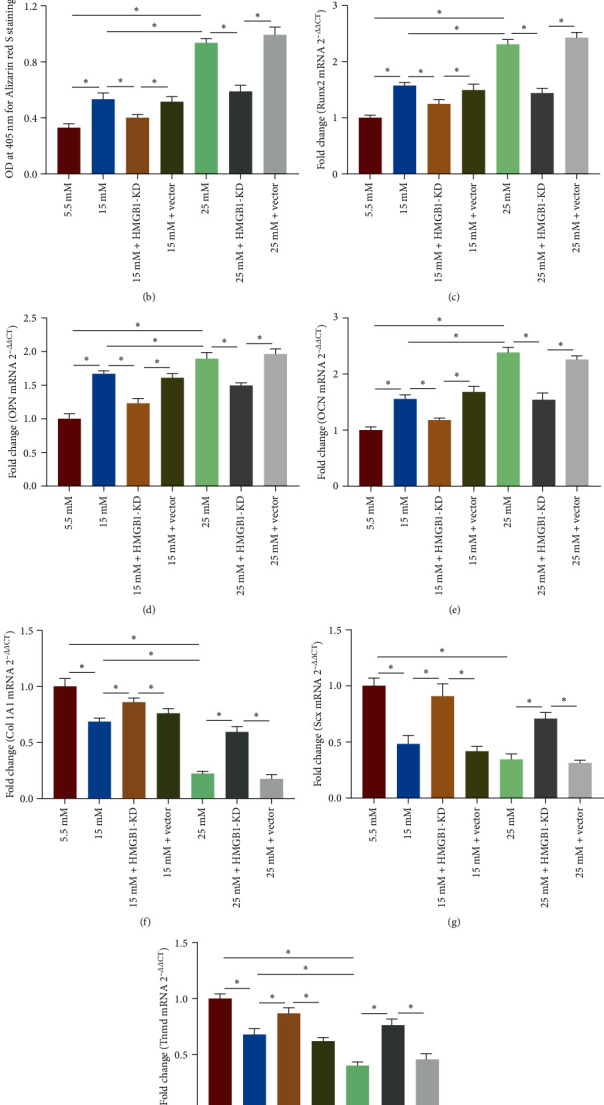
HMGB1 knockdown mitigates high glucose-induced erroneous differentiation of TSPCs. (a) Representative images of ARS staining of TSPCs after HMGB1 knockdown during osteogenic differentiation. (b) Quantification of ARS bound to the calcium nodules in TSPCs. (c–e) Relative mRNA levels of osteogenic genes in TSPCs after HMGB1 knockdown during osteogenic differentiation by qRT-PCR. (f–h) Relative mRNA levels of tendon-related genes in TSPCs after HMGB1 knockdown by qRT-PCR. A–G × 1; F–N × 40. KD, knockdown; vector, negative control vector.  ^*∗*^*P*  < 0.05.

To further investigate whether HMGB1 is the key cytokine associated with the erroneous differentiation of TSPCs, we knocked down HMGB1 in TSPCs using lentivirus targeting HMGB1. The downregulation of HMGB1 was confirmed by qRT-PCR and western blot analysis (*Supplementary 3*). Next, the TSPCs were cultured in osteogenic medium with high glucose, followed by evaluating the osteogenic differentiation ability. The results showed that the calcium deposition was significantly decreased in TSPCs after HMGB1 knockdown compared with that without HMGB1 knockdown (Figures 4(a) and 4(b)). Moreover, the upregulation of osteogenic genes under high glucose was notably blunted in TSPCs after HMGB1 knockdown (Figure 4(c)– 4(e)). Intriguingly, HMGB1 knockdown could rescue high glucose-induced downregulation of tenogenic genes in TSPCs (Figure 4(f)–4(h)), suggesting that the tenogenic differentiation capacity of TSPCs was ameliorated. Together, these results further confirmed the critical role of HMGB1 in high glucose-induced erroneous differentiation of TSPCs.

### 3.5. An Increase in HMGB1 Activates RAGE/*β*-Catenin Signaling

**Figure 5:**
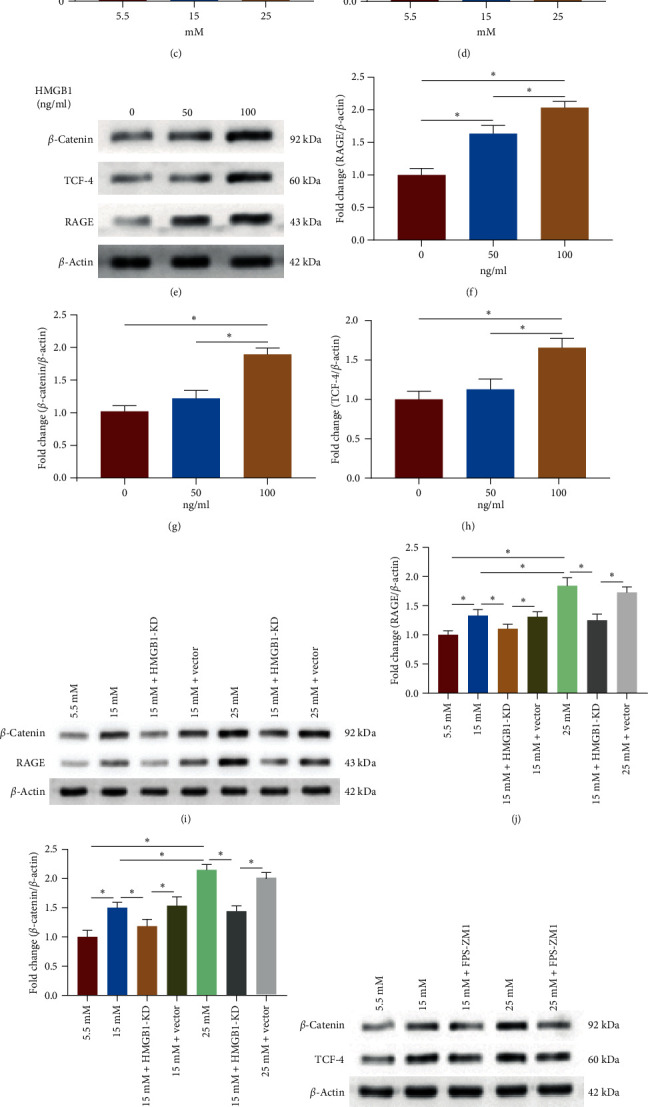
Increased HMGB1 activates the RAGE/*β*-catenin signaling pathway. (a–d) Western blot analysis of RAGE, *β*-catenin, and TCF-4 expression in TSPCs during osteogenic induction under normal and high glucose. (e–h) Western blot analysis of RAGE, *β*-catenin, and TCF-4 expression in TSPCs during osteogenic induction with different concentrations of recombinant HMGB1 (0, 50, and 100 ng/ml). (i–k) Western blot analysis of RAGE and *β*-catenin expression in TSPCs after HMGB1 knockdown during osteogenic induction. (l–n) Western blot analysis of *β*-catenin and TCF-4 expression in TSPCs treated with the RAGE inhibitor FPS-ZM1 (10 *μ*M) during osteogenic induction. KD, knockdown; vector, negative control vector.  ^*∗*^*P*  < 0.05.

To explore the underlying mechanism of the HMGB1-mediated erroneous differentiation of TSPCs, we examined the activation of RAGE/*β*-catenin signaling. The results showed that the expression of RAGE, *β*-catenin, and TCF-4 was dramatically increased in TSPCs during osteogenic differentiation under high glucose compared to that under normal glucose, indicating the activation of RAGE/*β*-catenin signaling (Figure 5(a)–5(d)). In addition, exogenous HMGB1 significantly increased the protein expression of RAGE, *β*-catenin, and TCF-4 in TSPCs during osteogenic induction (Figure 5(e)–5(h)). However, the expression of RAGE and *β*-catenin in TSPCs was remarkably reduced after HMGB1 knockdown during osteogenic differentiation under high glucose (Figure 5(i)–5(k)). To investigate the effect of RAGE activation on intracellular *β*-catenin signaling during osteogenic differentiation of TSPCs, we treated TSPCs with the RAGE inhibitor FPS-ZM1. The results showed that FPS-ZM1 treatment notably reduced the expression of *β*-catenin and TCF-4 during osteogenic differentiation of TSPCs under high glucose (Figure 5(l)–5(n)), suggesting that inhibition of RAGE could suppress the activity of downstream *β*-catenin signaling. Collectively, these results indicated that increased HMGB1 activated the RAGE/*β*-catenin signaling in TSPCs during osteogenic differentiation under high glucose, which might be associated with high glucose-induced erroneous differentiation of TSPCs.

### 3.6. Inhibition of RAGE/*β*-Catenin Signaling Ameliorates High Glucose-Induced Erroneous Differentiation of TSPCs

**Figure 6:**
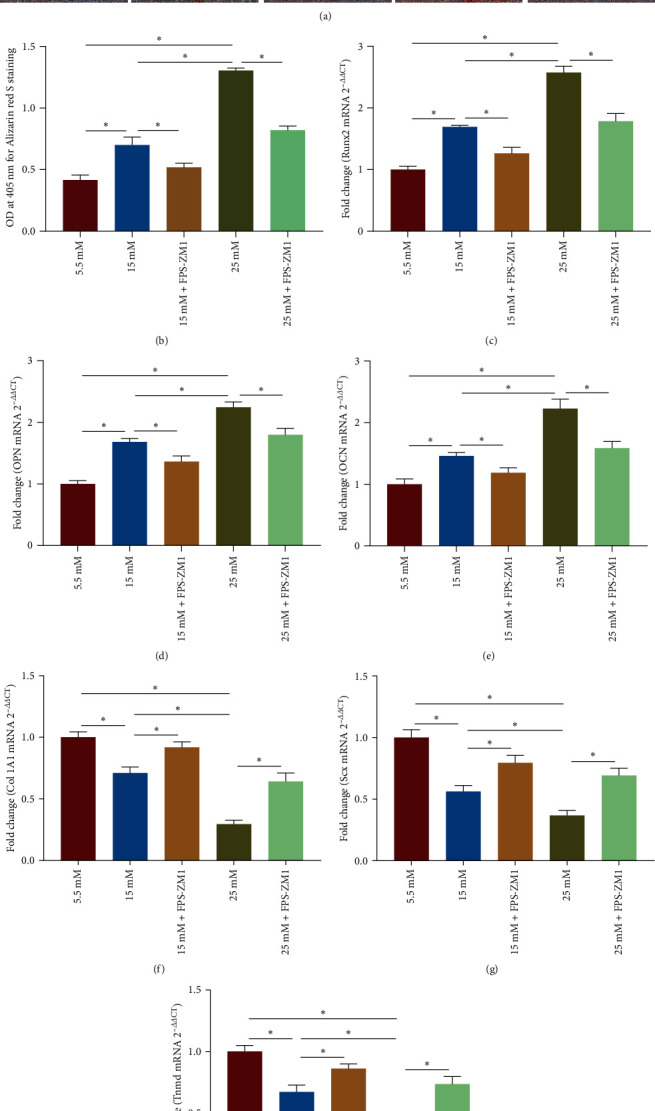
Inhibition of RAGE ameliorates high glucose-induced erroneous differentiation of TSPCs. (a) Representative images of ARS staining of TSPCs cultured with FPS-ZM1 (10 *μ*M) during osteogenic induction under high glucose. (b) Quantification of ARS bound to the calcium nodules in TSPCs cultured with FPS-ZM1. (c–e) Relative mRNA levels of osteogenic genes in TSPCs cultured with FPS-ZM1 during osteogenic induction by qRT-PCR. (f–h) Relative mRNA levels of tendon-related genes in TSPCs cultured with FPS-ZM1 by qRT-PCR. A–E × 1; F–J × 40.  ^*∗*^*P*  < 0.05.

**Figure 7:**
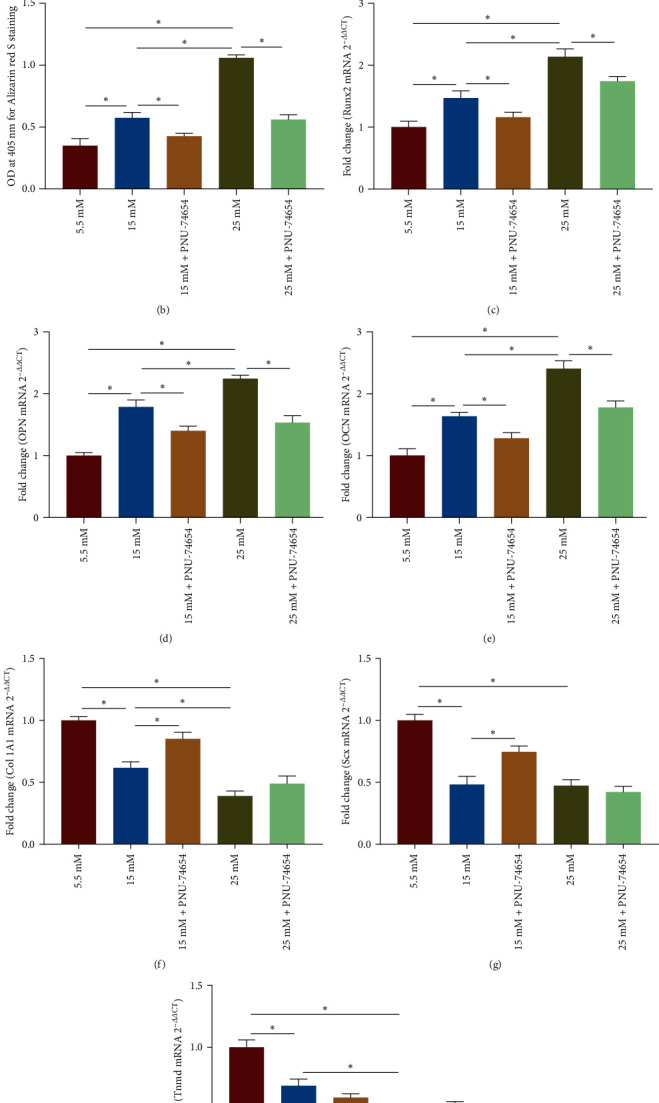
Inhibition of *β*-catenin signaling mitigates high glucose-induced erroneous differentiation of TSPCs. (a) Representative images of ARS staining of TSPCs cultured with the *β*-catenin inhibitor PNU-74654 (50 *μ*M) during osteogenic induction under high glucose. (b) Quantification of ARS bound to the calcium nodules in TSPCs cultured with PNU-74654. (c–e) Relative mRNA levels of osteogenic genes in TSPCs cultured with PNU-74654 during osteogenic induction by qRT-PCR. (f–h) Relative mRNA levels of tendon-related genes in TSPCs cultured with PNU-74654 by qRT-PCR. A–E × 1; F–J × 40.  ^*∗*^*P*  < 0.05.

To further investigate whether the regulation of high glucose-induced erroneous differentiation of TSPCs by HMGB1 depends on the activation of RAGE/*β*-catenin signaling, we treated TSPCs with the RAGE inhibitor FPS-ZM1 and the *β*-catenin inhibitor PNU-74654, respectively. Notably, FPS-ZM1 treatment decreased calcium deposition in TSPCs during osteogenic induction under high glucose (Figures 6(a) and 6(b)). Moreover, FPS-ZM1 treatment significantly reduced the mRNA expression of the osteogenic genes Runx2, OPC, and OCN in TSPCs during osteogenic differentiation under high glucose (Figure 6(c)–6(e)). Moreover, FPS-ZM1 treatment could rescue the decreased levels of the tenogenic markers Col IA1, Scx, and Tnmd in TSPCs under high glucose (Figure 6(f)–6(h)). In addition, inhibition of *β*-catenin signaling by PNU-74654 significantly reduced the intensity of ARS staining in TSPCs during osteogenic differentiation under high glucose (Figures 7(a) and 7(b)). The mRNA expression of osteogenic markers was also significantly decreased in TSPCs treated with PNU-74654 (Figure 7(c)–7(e)). Notably, PNU-74654 treatment could rescue the reduced mRNA expression of the tenogenic genes Col IA1 and Scx in TSPCs cultured in 15 mM of high glucose but not in 25 mM of high glucose (Figure 7(f)–7(h)). Taken together, these results further suggested that the RAGE/*β*-catenin signaling pathway played an essential role in regulating high glucose-induced erroneous differentiation of TSPCs.

## 4. Discussion

Diabetic tendinopathy is a common musculoskeletal disorder in individuals with diabetes and has been linked to the impaired function of TSPCs. TSPCs exhibit the self-renewal capacity and multidifferentiation potential like MSCs and play a critical role in tendon repair, regeneration, and homeostasis [10, 11]. A recent study using single-cell transcriptomics identified the Tppp3/Pdgfra-positive cell population as TSPCs, which could generate new tenocytes and undergo self-renewal upon tenon injury [36]. Yin et al. [37] reported that, compared with nestin-negative TSPCs, nestin-positive TSPCs exhibited a superior self-renewal and tenogenic differentiation potentiality, which was involved in the development and endogenous repair of tendon tissues. However, our previous studies have demonstrated that TSPCs isolated from diabetic rat tendons exhibit reduced proliferation ability, enhanced osteo-chondrogenic differentiation potential, and decreased tenogenic differentiation ability, which is involved in the pathogenesis of diabetic tendinopathy [5]. Hyperglycemia is a typical manifestation of diabetes mellitus and a critical pathogenic factor for diabetes-related complications, including musculoskeletal diseases and tendinopathy [7, 8]. A high glucose environment can lead to decreased viability, cell death, altered fate, and impaired function of TSPCs [6, 14, 38, 39]. Studies have reported that the expression of tenogenic differentiation markers is reduced while the expression of osteochondrogenic differentiation markers is increased in TSPCs treated with high glucose [6, 14]. In this study, high glucose treatment in vitro promoted the osteogenic differentiation of TSPCs. In addition, consistent with the previous studies, our study showed downregulated expression of tenogenic differentiation genes and upregulated expression of osteogenic differentiation genes in TSPCs under high glucose treatment. These results suggested the impaired tenogenic differentiation ability and promoted osteogenic differentiation capacity of TSPCs under high glucose, indicating the erroneous differentiation. A hyperglycemic microenvironment may affect tendon healing by dysregulating the behavior of resident stem cells, contributing to the pathogenesis of diabetic tendinopathy. Rui et al. [40, 41] proposed the theory of erroneous differentiation of TSPCs for the first time and elucidated its potential role in the pathogenesis of tendinopathy. Tendon calcification is a common occurrence of tendinopathy in patients with diabetes [42, 43], but the mechanism of tendon calcification has not yet been fully illuminated. TSPCs are superior for tendon homeostasis maintenance and repair due to their tendon tissue-specific differentiation properties [11]. The spontaneous tenogenic differentiation has been reported to be a specific feature of TSPCs [44]. However, studies reported that the enhanced osteogenic differentiation potential of TSPCs contributed to increased calcification in tendons [45]. Feng et al. [46] showed that a subpopulation of TSPCs expressing Cathepsin K was the primary source of cells involved in tendon calcification. Therefore, the erroneous differentiation of TSPCs may account for the increased expression of osteogenic genes and reduced expression of tendon-related genes and calcification in diabetic tendons. The altered fate of TSPCs is likely to affect tendon components and homeostasis, jeopardize mechanical properties, and impair tendon repair and regeneration capacity, facilitating the development and process of diabetic tendinopathy.

Here, we report for the first time that HMGB1 expression is closely related to the erroneous differentiation of TSPCs. HMGB1 is widely expressed and resides primarily in the nucleus, where it regulates transcription, replication, recombination, and genome stability in various physiological and pathological processes [15, 47]. Upon tissue injury or cellular stress, HMGB1 can be translocated into the cytoplasm and even secreted into the extracellular environment passively or actively [15, 47]. The different functions of HMGB1 vary with distributions, and extracellular HMGB1 is involved in tissue injury and regeneration as a DAMP or alarmin [26]. Previous studies have reported that HMGB1 is increased in diabetic patients and is involved in the pathogenesis of many diabetes-related diseases [19, 20]. In recent years, accumulating evidence has shown that HMGB1 plays crucial roles in the pathogenesis of tendinopathy [26]. However, the role of HMGB1 in TSPCs has rarely been investigated. Previous studies reported that high glucose treatment increased the expression of HMGB1 in MSCs [48] and human retinal endothelial cells [49]. Our results showed that high glucose treatment significantly increased the expression of HMGB1 in TSPCs. Moreover, HMGB1 was localized primarily in the nucleus of TSPCs, whereas the level of HMGB1 was remarkably decreased in the nucleus and significantly increased in the cytoplasm under high glucose treatment. In addition, the concentration of HMGB1 in the supernatant of TSPCs was elevated under high glucose treatment. These results indicate that high glucose treatment may promote the translocation of HMGB1 from the nucleus to the cytoplasm in TSPCs and further secretion. However, what drives the expression and secretion of HMGB1 under high glucose treatment needs to be further investigated. A recent study reported that the translocation of HMGB1 from the nucleus to the cytoplasm, which functions as an autophagy promoter, was associated with the activation of autophagy in TSPCs [50]. However, extracellular HMGB1 is a novel cytokine involved in inflammation, tissue damage, and regeneration [51]. Recent studies have indicated that extracellular HMGB1 is also involved in the regulation of stem cells. Exogenous HMGB1 could inhibit the proliferation and promote the migration and osteogenic differentiation of human MSCs [27, 28, 51]. Our results showed that exogenous HMGB1 promoted the osteogenic differentiation ability and reduced the tenogenic differentiation ability of TSPCs, and HMGB1 knockdown attenuated high glucose-induced erroneous differentiation of TSPCs. Collectively, these results indicate that HMGB1 plays a critical role in the erroneous differentiation of TSPCs, suggesting that HMGB1 is a potential therapeutic target for diabetic tendinopathy.

HMGB1 can regulate the differentiation of stem cells by binding with RAGE [16]. Sun et al. [52] reported that high glucose treatment could increase the expression of RAGE in BMSCs. Moreover, treatment with HMGB1 remarkably increased the expression of RAGE in human BMSCs during osteogenic differentiation [27]. In addition, *β*-catenin interacts with TCF-4 in the nucleus to activate the transcription of osteogenic genes and has emerged as a critical regulator of the osteogenic differentiation of stem cells [30–32]. Increased RAGE could promote osteogenic differentiation of vascular smooth muscle cells and calcification via modulating downstream *β*-catenin signaling [53, 54]. Our results showed that the RAGE/*β*-catenin signaling pathway was activated in TSPCs during osteogenic differentiation under high glucose. Exogenous HMGB1 significantly increased the expression of RAGE and *β*-catenin, and HMGB1 knockdown inhibited their expression. Besides, inhibition of RAGE attenuated the activity of *β*-catenin signaling. These results demonstrated that an increase in HMGB1 activated the RAGE/*β*-catenin pathway under high glucose treatment. Interestingly, inhibition of RAGE/*β*-catenin signaling could mitigate high glucose promoted osteogenic differentiation of TSPCs and partially rescue the tenogenic differentiation ability. Therefore, HMGB1 mediates high glucose-induced erroneous differentiation of TSPCs via the RAGE/*β*-catenin pathway, and blockade of RAGE/*β*-catenin signaling could ameliorate high glucose-induced erroneous differentiation of TSPCs.

A limitation in the current study is that high glucose treatment in vitro does not completely mimic the diabetic or hyperglycemic microenvironment in vivo. Diabetes is a systemic metabolic disease. Some major pathophysiological hallmarks of diabetes, such as hyperglycemia, the accumulation of advanced glycation end products (AGEs), obesity, and insulin resistance, may contribute to tendinopathy individually and synergistically [8]. The factors associated with diabetes, such as the accumulation of AGEs [9], adipokines [55], insulin [56], and adiponectin [57], may also affect the function of TSPCs, contributing to the development of diabetic tendinopathy. Here, we mainly focused on the underlying effect of a high glucose environment on TSPCs. Besides, although some studies have reported the identification of TSPC populations using single-cell transcriptomics [36] and have analyzed the subpopulations of TSPCs that exhibit superior tenogenic differentiation potentiality or are involved in tendon ossification [37, 46], further in vivo confirmation that the nontenogenic changes in diabetic tendons are derived from the erroneous differentiation of TSPCs is needed. Which subpopulation of TSPCs plays critical roles in diabetic tendinopathy also requires further investigation. Additionally, there may also be other signaling pathways regulating TSPC differentiation. Our results showed that the *β*-catenin signaling primarily plays a significant role in regulating the osteogenic differentiation of TSPCs. There may be other signaling pathways that regulate the tenogenic differentiation of TSPCs in diabetic tendinopathy, which needs to be further explored.

## 5. Conclusions

**Figure 8:**
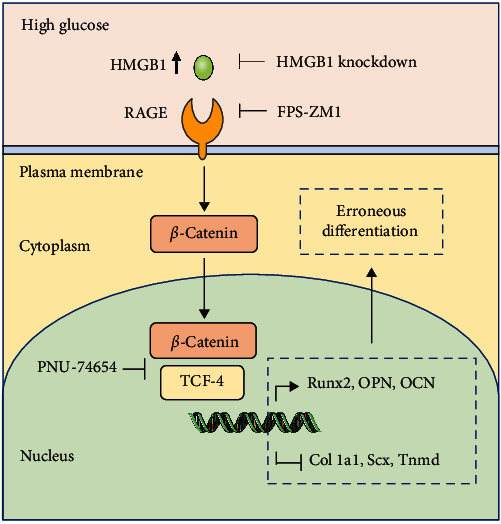
Schematic model of the proposed mechanism of erroneous differentiation of TSPCs under high glucose. Under high glucose conditions, increased HMGB1 activates the RAGE/*β*-catenin signaling pathway, leading to enhanced osteogenic differentiation of TSPCs and decreased tenogenic differentiation, contributing to the pathogenesis of diabetic tendinopathy.

In conclusion, our study demonstrated that high glucose treatment promoted the osteogenic differentiation and inhibited the tenogenic differentiation of TSPCs, and increased the expression and secretion of HMGB1 in TSPCs. HMGB1 is a critical cytokine which plays a significant role in high glucose-induced erroneous differentiation of TSPCs. Moreover, we suggest that HMGB1 regulates high glucose-induced erroneous differentiation of TSPCs through activating the RAGE/*β*-catenin signaling pathway (Figure 8). Our findings suggest a novel essential mechanism for the erroneous differentiation of TSPCs, which might contribute to the pathogenesis of diabetic tendinopathy and provide a promising therapeutic target and approach for diabetic tendinopathy.

## Data Availability

The data presented in the study are available upon reasonable request from the corresponding authors.

## Supplementary Materials

Supplementary 1 Primer sequences for qRT-PCR.

Supplementary 2 Antibodies.

Supplementary 3 Knockdown of HMGB1 in TSPCs.
