# Supplementary material for: HMGB1 Modulates High Glucose-Induced Erroneous Differentiation of Tendon Stem/Progenitor Cells through RAGE/β-Catenin Pathway
Source: Stem Cells Int. 2024 Apr 9;2024:2335270. doi: 10.1155/2024/2335270 (PMC11022503; doi:10.1155/2024/2335270)
Supplement: Supplementary 1 — Primer sequences for qRT-PCR. [file 2335270.f1.docx]

**Table S1.** Primer sequences for qRT-PCR.

| Target gene | Forward | Reverse |
| --- | --- | --- |
| Runx2 | 5′-CCGATGGGACCGTGGTT-3′ | 5′-CAGCAGAGGCATTTCGTAGCT-3′ |
| OPN | 5′-TCCAAGGAGTATAAGCAGCGGGCCA-3′ | 5′-CTCTTAGGGTCTAGGACTAGCTTCT-3′ |
| OCN | 5′-GGTGCAAAGCCCAGCGACTCT-3′ | 5′-GGAAGCCAATGTGGTCCGCTA-3′ |
| Col1A1 | 5′-AGAGGCATAAAGGGTCATCGTG-3′ | 5′-AGACCGTTGAGTCCATCTTTGC-3′ |
| Scx | 5′-CGAGAACACCCAGCCCAAAC-3′ | 5′-CGTCTTTCTGTCACGGTCTTTG-3′ |
| Tnmd | 5′-GACCTATGGCATGGAGCACAC-3′ | 5′-TGTTTCATCGGTGCCATTTCC-3′ |
| HMGB1 | 5′-AATGTATCCCCAAAAGCGT-3′ | 5′-TAGATAGAAGGGCAAAGGCA-3′ |
| β-actin | 5′-ATCGTGGGCCGCCCTAGGCA-3′ | 5′-TGGCCTTAGGGTTCAGAGGGG-3′ |
