# Supplementary material for: HMGB1 Modulates High Glucose-Induced Erroneous Differentiation of Tendon Stem/Progenitor Cells through RAGE/β-Catenin Pathway
Source: Stem Cells Int. 2024 Apr 9;2024:2335270. doi: 10.1155/2024/2335270 (PMC11022503; doi:10.1155/2024/2335270)
Supplement: Supplementary 2 — Antibodies. [file 2335270.f2.docx]

**Table S2.** Antibodies.

| Product name | Source catalog | Application |
| --- | --- | --- |
| Anti-HMGB1 antibody | Proteintech (10829-1-AP) | WB/IF |
| Anti-β-actin antibody | Proteintech (20536-1-AP) | WB |
| Anti-Lamin B1 antibody | Proteintech (12987-1-AP) | WB |
| Anti-β-catenin antibody | Cell Signaling Technology (#8480) | WB |
| Anti-TCF-4 antibody | Santa Cruz Biotechnology (sc-166699) | WB |
| Anti-RAGE antibody | Proteintech (16346-1-AP) | WB |
