# Supplementary material for: HMGB1 Modulates High Glucose-Induced Erroneous Differentiation of Tendon Stem/Progenitor Cells through RAGE/β-Catenin Pathway
Source: Stem Cells Int. 2024 Apr 9;2024:2335270. doi: 10.1155/2024/2335270 (PMC11022503; doi:10.1155/2024/2335270)
Supplement: Supplementary 3 — Knockdown of HMGB1 in TSPCs. [file 2335270.f3.docx]

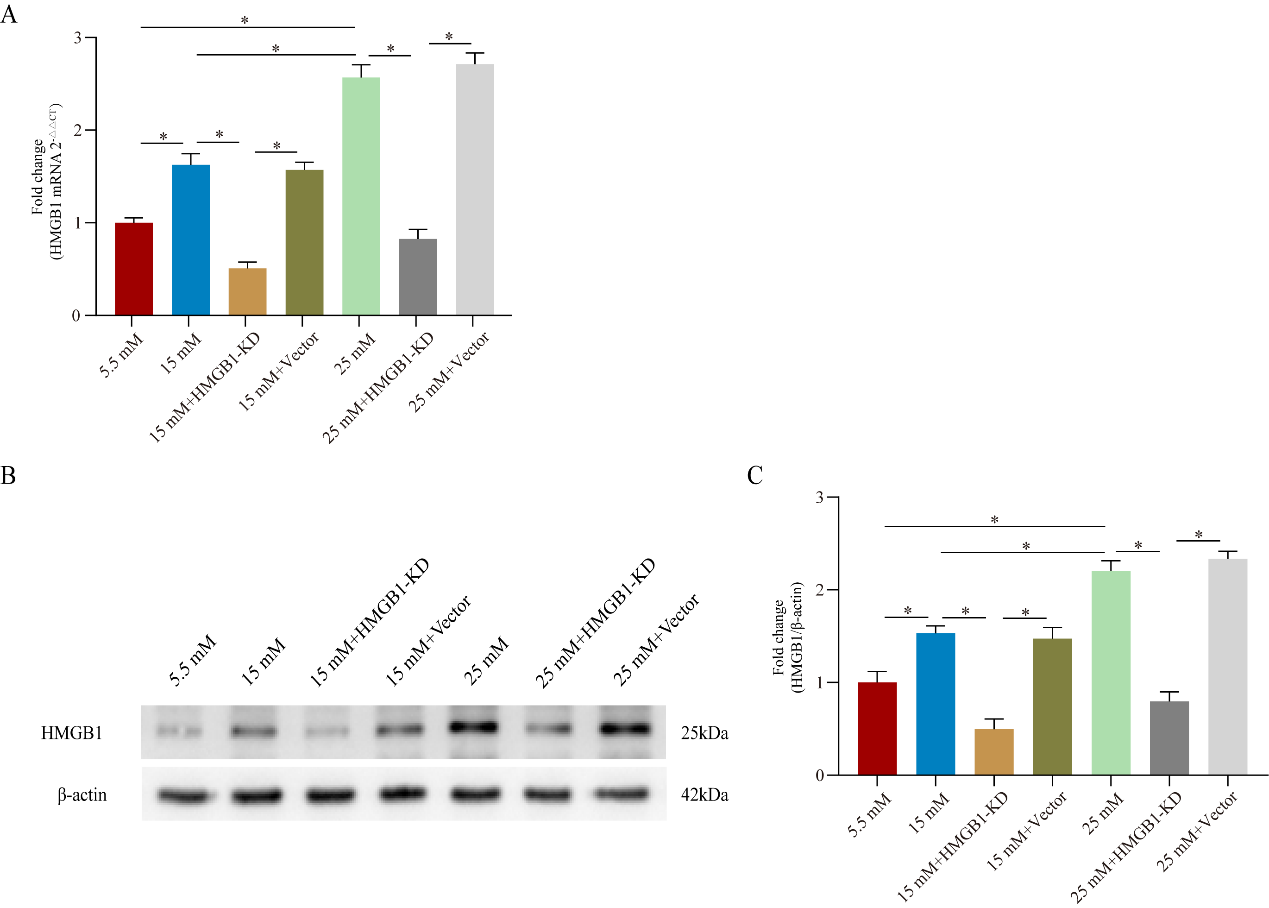


**Fig. S1** Knockdown of HMGB1 in TSPCs. **(A)** qRT-PCR analysis of relative mRNA expression of HMGB1 in TSPCs after HMGB1 knockdown. **(B, C)** Western blot analysis of HMGB1 expression in TSPCs after HMGB1 knockdown. KD: knockdown; Vector: negative control vector. *P < 0.05.
